# Study on Influencing Factors Analysis of Gastric Tube Insertion Length and Construction of Estimation Method

**DOI:** 10.3389/fsurg.2022.942881

**Published:** 2022-07-11

**Authors:** Hua Zhang, Huaqin Wang, Xiaoyu Fan, Xiangqun Cao, Wan Su, Bo Yang

**Affiliations:** ^1^ Department of Gastroenterology, The Second Xiangya Hospital, Central South University, Changsha, Hunan, China; ^2^ Clinical Nursing Teaching and Research Section, The Second Xiangya Hospital, Central South University, Changsha, Hunan, China; ^3^ Department of Critical Care Medicine, The Second Xiangya Hospital, Central South University, Changsha, Hunan, China; ^4^ Medical College, Jishou University, Jishou, Hunan, China

**Keywords:** gastrointestinal decompression, gastric tube insertion, influencing factors, estimation method, insertion length

## Abstract

**Background:**

Influenced by individual differences, the depth of gastric tube placement is often different. Clinically, it is necessary to seek a simple and accurate gastric tube insertion scheme to improve the clinical efficacy of indwelling gastric tube.

**Materials and Methods:**

A total of 100 adult patients undergoing transesophageal manometry *via* nose were included in the study. The *in vivo* length (NCL) of apex-cardia was measured. At the same time, we entered our institutional database, summarized the clinical data of 100 patients, and analyzed the risk factors affecting NCL using stepwise regression analysis.

**Results:**

The NCL length scores of patients with different gender, age, marital status, height, weight, BMI, sitting height, sternum length, hairline-xiphoid process, nose tip-earlobe-xiphoid process and earlobe-xiphoid process were statistically significant (*P* < 0.05). Height, sitting height, gender, BMI and earlobe-xiphoid process were the factors that affected the NCL length score (*P* < 0.05). The prediction equation of the estimation method of gastric tube insertion length was as follows: NCL length score = 39.907 + 2.909× height +0.865× sitting height. Adjust *R*^2^ to 0.506. NCL was positively correlated with height and sitting height. Among them, the correlation with height (*r *= 0.711, *P *< 0.001) and sitting height (*r *= 0.397, *P *< 0.001).

**Conclusion:**

Height, sitting height, gender, BMI and earlobe-xiphoid process were the factors that affected the score of NCL length. There was a significant positive correlation between height, sitting height and NCL length. On this basis, the length of nasogastric tube insertion could be estimated.

## Introduction

Refers to inserting the catheter into the gastrointestinal tract through the nasal cavity or oral cavity, and providing the patients with the necessary food, nutrient solution, water and drugs through the catheter, or performing gastric lavage and gastrointestinal decompression through the catheter, which is a routine nursing operation with wide clinical application (1, 2). Indwelling gastric tube can not only supply the necessary nutrition for clinical patients, but also achieve the effect of gastrointestinal decompression. Compared with foreign countries, only a small number of studies in China have explored the factors affecting the length of gastric tube placement in adults and its prediction equation through correlation or regression analysis. The existing guidelines also fail to address such issues as the specific length to be extended of gastric tube insertion, and whether it is safe to extend the insertion length. Although the length from the hairline to the xiphoid process or from the earlobe to the xiphoid process is often used as the insertion depth in clinic, the insertion depth is often not the same due to individual differences of patients, so the accuracy of this method has always been questioned (3, 4). Therefore, in this study, the *in vivo* length (NCL) of nasal tip–cardia in adults used as a dependent variable to explore its relationship with other clinical data such as gender, age, height, and weight. At the same time, the prediction equation with optimal stability and accuracy was established based on the above results, in order to provide a theoretical basis for the estimation of the length of gastric tube placement.

## Materials and Methods

### General Information

The convenience sampling method was used to select adult patients who required transesophageal manometry *via* nose or gastroscopy *via* mouth in a 3A hospital in Changsha from March 2020 to February 2022.

Inclusion criteria: (1) Age ≥ 18 years old. (2) Conscious, willing to undergo gastroscopy or esophageal manometry, and willing to participate in this study. (3) There is no obvious thoracic deformity, spinal deformity and developmental abnormality. (4) There is no previous operation history of upper digestive tract such as esophagus and stomach. (5) Patients with the course of disease less than three months.

Exclusion criteria: (1) Patients whose esophageal dentate line was found to be vague by gastroscopy. (2) Patients who had obvious vomiting during gastroscopy and failed to complete gastroscopy. (3) Gastroscopy shows esophageal abnormalities, affecting the measurement of esophageal length. (4) esophageal manometry shows severe disorder of esophageal movement. (5) Those who cannot lie flat or sit upright due to scleroderma, neck disease or other reasons.

This study was approved by the Hospital Ethics Committee with the patient's consent and informed consent form signed.

### Research Method

The length of gastric tube insertion was the *in vivo* length from the tip of the nose to the cardia. The manometer was placed into the esophagus through the nasal cavity, and the position of the front end of the manometer in the esophagus was determined according to the waveform obtained from the pressure measurement. Read the scale when the front end of the manometer reaches the junction of esophagus and stomach. This distance is the *in vivo* length of nasal tip–cardia, and record as NCL (5).

Clinical data of all patients were collected, including gender, age, marital status, height, weight, BMI of the patients before gastric tube insertion, whether the gastric tube was indwelling for the first time, sitting height, sternal length, diagnosis, chest circumference, waist circumference, hip circumference, xiphoid process-navel length, hairline-xiphoid process, nasal tip-earlobe-xiphoid process, earlobe-xiphoid process and the material of gastric tube.

### Statistical Method

SPSS 21.0 is used to establish the database and analyze the data, and the measurement data is described by $\left(\bar{x}\pm s\right)$. Count data use cases and percentage descriptions, and compare between groups by independent sample *t* test and one-way ANOVA. Pearson correlation analysis was used to analyze the correlation between NCL length score, height and sitting height. The variables with statistically significant differences between groups after *t*-test and Chi-square test were used as independent variables, and stepwise regression linear analysis was carried out, and the estimation equation of gastric tube insertion length was established. All analyses are based on 95% confidence intervals. *P *< 0.05 is statistically significant.

## Result

### General Information

A total of 100 patients were included according to the inclusion and exclusion criteria of research subjects. There 27 females and 73 males. The mean age was (44.27 ± 14.86) years old. There were 55 cases of achalasia of cardia, 7 cases of abdominal pain, 16 cases of Crohn's disease, 9 cases of ulcerative colitis, 8 cases of dysphagia and 5 cases of gastrointestinal bleeding.

### Single Factor Analysis of NCL Length Score

As shown in Table 1, the scores of NCL length among patients with different gender, age, marital status, height, weight, BMI, sitting height, sternal length, the length from hairline to xiphoid process, the length of nasal tip–earlobe–xiphoid process, and the length from earlobe to xiphoid process were statistically significant (*P* < 0.05). The scores of NCL length among patients with different diagnoses, the first indwelling gastric tube, history of hypertension, chest circumference, waist circumference, hip circumference, and the length from xiphoid process to navel, and gastric tube material were not significant (*P* > 0.05).

**Table 1:** Single factor analysis of NCL length score.

Clinical features	*n*	Constituent ratio (%)	NCL (score)	*t*/*F*	*P*
Gender					
Female	27	27.00	45.57 ± 2.77	3.624	0.001
Male	73	73.00	48.55 ± 3.92		
Age (years)					
≤27	18	18.00	45.68 ± 4.29	2.53	0.045
28–39	23	23.00	49.07 ± 3.56		
40–49	21	21.00	47.26 ± 3.33		
50–59	23	23.00	48.66 ± 4.15		
60–78	15	15.00	47.44 ± 3.24		
Marital status					
Married	82	82.00	48.19 ± 3.64	2.564	0.012
Unmarried	18	18.00	45.68 ± 4.29		
Disease type					
Cardiac achalasia	55	55.00	47.80 ± 3.94	1.05	0.392
Abdominalgia	7	7.00	44.84 ± 2.48		
Crohn's disease	16	16.00	48.79 ± 4.81		
Ulcerative colitis	9	9.00	47.64 ± 3.23		
Dysphagia	8	8.00	48.24 ± 2.58		
Gastrointestinal bleeding	5	5.00	47.10 ± 4.83		
First indwelling gastric tube					
Yes	79	79.00	47.51 ± 3.76	0.329	0.745
No	21	21.00	47.80 ± 2.84		
History of hypertension					
Yes	38	38.00	47.91 ± 3.74	1.400	0.165
No	62	62.00	46.86 ± 3.58		
Height (cm)					
≤160	32	32.00	44.74 ± 2.88	28.53	<0.001
161–170	42	42.00	47.62 ± 3.23		
171–180	22	22.00	51.30 ± 1.99		
181–183	4	4.00	53.45 ± 2.21		
Weight (kg)					
≤50	28	28.00	45.76 ± 2.72	5.66	0.001
51–59	21	21.00	47.10 ± 4.05		
60–69	38	38.00	49.20 ± 3.32		
70–106	13	13.00	48.78 ± 5.25		
BMI (kg/m^2^)					
≤25	61	61.00	45.52 ± 3.16	9.569	0.002
>25	39	39.00	51.22 ± 2.45		
Chest measurement (cm)					
≤80	20	20.00	46.57 ± 4.08	1.49	0.223
81–89	43	43.00	48.40 ± 3.44		
90–99	33	33.00	47.36 ± 4.32		
100–112	4	4.00	49.67 ± 1.07		
Waistline (cm)					
≤70	27	27.00	46.57 ± 3.86	1.64	0.185
71–79	31	31.00	48.59 ± 3.49		
80–89	31	31.00	47.55 ± 4.11		
90–112	11	11.00	48.77 ± 3.83		
Hipline (cm)					
≤80	12	12.00	47.70 ± 3.14	1.85	0.143
81–89	39	39.00	46.83 ± 3.87		
90–99	45	45.00	48.28 ± 4.00		
100–115	4	4.00	50.73 ± 2.54		
Sitting height (cm)					
≤80	16	16.00	45.57 ± 3.75	11.78	<0.001
81–89	42	42.00	46.47 ± 4.15		
90–99	28	28.00	50.75 ± 1.91		
100–137	14	14.00	48.04 ± 2.25		
Sternal length (cm)					
≤15	11	11.00	45.72 ± 5.19	3.09	0.031
16–19	44	44.00	47.30 ± 3.60		
20–21	25	25.00	47.85 ± 3.67		
22–28	20	20.00	49.70 ± 3.27		
The length from xiphoid process to navel (cm)					
≤15	23	23.00	47.09 ± 2.88	0.64	0.529
16–20	70	70.00	47.85 ± 3.82		
21–47	7	7.00	48.86 ± 6.77		
The length from hairline to xiphoid process (cm)					
≤45	33	33.00	46.06 ± 3.83	5.72	0.005
46–55	59	59.00	48.39 ± 3.62		
56–59	8	8.00	49.93 ± 3.72		
The length of nasal tip–earlobe–xiphoid process (cm)					
≤50	47	47.00	45.98 ± 3.67	11.45	<0.001
51–55	43	43.00	49.14 ± 3.33		
56–64	10	10.00	50.03 ± 3.50		
The length from earlobe to xiphoid process (cm)					
≤35	40	40.00	45.46 ± 3.73	15.27	<0.001
36–38	38	38.00	48.99 ± 3.30		
39–47	22	22.00	49.74 ± 2.95		
Gastric tube material					
Silica gel	57	57.00	45.17 ± 2.98	1.65	0.197
Fukai gastric tube	31	31.00	46.28 ± 2.41		
Improved gastric tube	12	12.00	45.85 ± 2.69		

### Variable Assignment

The length of NCL is always divided into dependent variables, and the items in general data that affect the length of NCL are included in the regression equation as independent variables. Independent variable assignment is shown in Table 2.

**Table 2:** Assignment of influencing factors of NCL length score.

Variable	Assignment
Dependent variable	
NCL	Enter as actual value.
Independent variable	
Gender	Female = 1, Male = 2
Age (years)	≤27 = 1,28–39 = 2,40–49 = 3,50–59 = 4,60–78 = 5
Marital status	Married = 1,Unmarried = 2
Height (cm)	≤160 = 1,161–170 = 2,171–180 = 3,181–183 = 4
Weight (kg)	≤50 = 1,51–59 = 2,60–9 = 3,70–106 = 4
BMI (kg/㎡)	≤25 = 1,>25 = 2
Sitting height (cm)	≤80 = 1,81–9 = 2,90–99 = 3,100–137 = 4
Sternal length (cm)	≤15 = 1,16–19 = 2,20–21 = 3,22–28 = 4
The length from hairline to xiphoid process (cm)	≤45 = 1,46–55 = 2,56–59 = 3
The length of nasal tip–earlobe–xiphoid process (cm)	≤50 = 1,51–55 = 2,56–64 = 3
The length from earlobe to xiphoid process (cm)	≤35 = 1,36–38 = 2,39–47 = 3

### Multiple Stepwise Linear Regression Analysis of Influencing Factors of NCL Length Score

As shown in Table 3, height, sitting height, sex, BMI and the length from earlobe to xiphoid process are the factors that affect NCL length score (*P* < 0.05). According to the regression principle, an equation is established. At the same time, meaningful variables are screened according to regression analysis. The prediction equation of the gastric tube insertion length estimation method in this study is established as follows: NCL length score = 39.907 + 2.909× height +0.865× sitting height. *R*^2^ is 0.506, and the regression equation can explain 50.6% variation degree of dependent variable.

**Table 3:** Multiple stepwise linear regression analysis of influencing factors of NCL length score.

Influencing factor	*B*	*SE*	*β*	*T*	*P*
Constant	39.907	0.911	_	43.782	0.000
Height	2.909	0.339	0.632	8.593	0.000
Sitting height	0.865	0.309	0.206	2.798	0.006
Gender	−1.803	0.675	−0.208	−2.673	0.009
Age	−0.104	−1.719	0.089	−0.175	0.896
Marital status	0.015	0.236	0.814	0.024	0.855
BMI	3.499	0.579	0.444	6.047	0.000
Sternal length	0.003	0.048	0.962	0.005	0.670
The length from hairline to xiphoid process	−0.165	−1.844	0.068	−0.187	0.408
The length of nasal tip–earlobe–xiphoid process	−0.176	−1.309	0.194	−0.134	0.184
The length from earlobe to xiphoid process	0.257	0.089	0.196	2.879	0.005

### Correlation Analysis Between Height, Sitting Height and NCL

**Figure 1:**
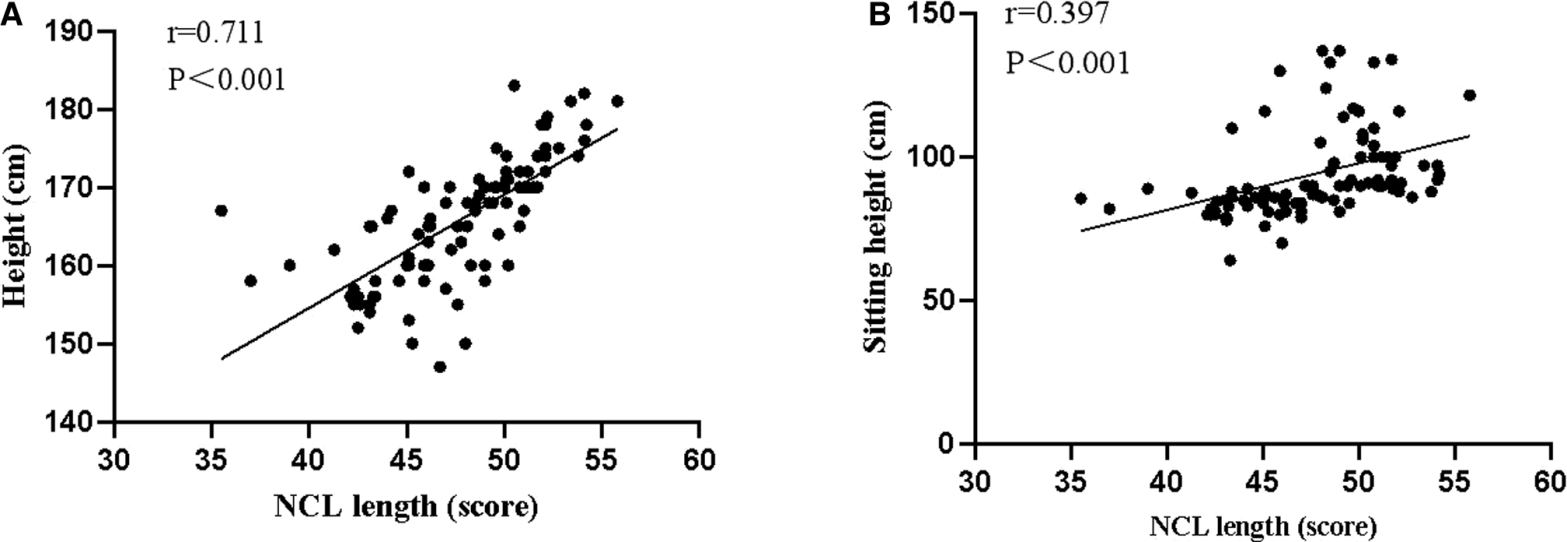
Scatter diagram for correlation analysis of NCL length score with height and sitting height ((**A**) Correlation analysis of NCL length score and height. (**B**) Correlation analysis of NCL length score and sitting height).

As shown in Table 4 and Figure 1, NCL was positively correlated with height and sitting height. Among them, the correlation with height (*r *= 0.711, *P *< 0.001) and sitting height (*r *= 0.397, *P *< 0.001).

**Table 4:** Correlation analysis of height, sitting height and NCL.

Project	NCL	
	*r*	*P*
Height	0.711	<0.001
Sitting height	0.397	<0.001

## Discussion

Gastric tube is a common drainage tube after surgery, its clinical effect has been widely recognized. The shape and size of the stomach are affected by such factors as the gastric volume, the stage of food digestion, the intestinal condition, and the body position. Therefore, although there are many studies on the estimation method of the insertion length of gastric tube in China and abroad, their views have not yet been unified. The lengths of gastric tube recommended by different institutes were different, and the common clinical values include 45–55 cm, 55–65 cm, 55–68 cm, and 55–70 cm (6–8). In addition, the commonly used body surface measurement methods for the length of gastric tube in clinic include the length from nasal tip to earlobe to xiphoid process (NEX), the length from nasal tip to earlobe to navel to the midpoint of xiphoid process (NEMU), the length from hairline to xiphoid process (FX), the length from hairline to navel (FU), and the length from hairline to navel to the midpoint of xiphoid process (FMU) (9–12). However, the determination of the above method is mainly based on the clinical experience of physicians and their observation of the treatment status quo, which is greatly affected by individual differences and has a large possibility of operation error.

The study found that if the actual length of gastric tube placement was taken as the dependent variable *Y*, the value of *Y* would not only be affected by such independent variables as height and weight as defined in the study, but also by the instability of its own endpoint. Moreover, the bias of self-endpoint is difficult to control, so it is difficult to establish a relatively stable prediction equation. Therefore, it is difficult to establish a relatively stable prediction equation clinically. Different from the front end of gastric tube, the right side of cardia is wrapped in lesser omentum together with the lower end of esophagus, the front and left sides were covered by peritoneum, and the back is diaphragm esophageal ligament Therefore, although the mobility of the stomach is great, the position of the cardia is relatively fixed (13). So when discussing the length of gastric tube insertion, converting the dependent variable *Y* to the *in vivo* length from the nasal tip to the cardia can reduce the bias caused by the movement of the dependent variable's own endpoint and is more conducive to establishing a relatively stable prediction equation. On this basis, NCL was replaced in this study by the *in vivo* length of the nasal tip-cardia.

It has been suggested that the accuracy of the inserted length is closely related to the prognosis of patients, so it is necessary to analyze the factors affecting the inserted length of gastric tube. The results of this study showed that height, sitting height, gender, BMI and the length from the earlobe to the xiphoid process were the factors that affected the NCL length score (*P *< 0.05), and height, sitting height and NCL had a positive correlation. Ellett et al. based on the accuracy of children of similar age to the length of gastric tube insertion by comparing their height (ARHB), the length from tip to earlobe to xiphoid process (NEX), and the length from tip to earlobe to navel (NEMU), and found that the height (ARHB) and the length from tip to earlobe to navel (NEMU) were more accurate than the length from tip to earlobe to xiphoid process (NEX) (14). Malta et al. explored the relationship between the distance from the incisors to the gastroesophageal junction (recorded as EGD), height, the distance from the earlobe to the xiphoid process (EX), and other measured values *in vitro*, and found that height was one of the independent variables with the strongest correlation with EGD (15). Meanwhile, studies have also shown that the height is directly proportional to the length of the gastric tube placement (16), which is similar to the results of this study. The NCL values of patients with different BMI have significant differences. As the height and weight of men are generally higher than those of women, it is believed that there should be differences between men and women in the intubation length (17). Meanwhile, with height and sitting height as independent variables and NCL length as dependent variable, the regression equation was derived: NCL length score = 39.907 + 2.909× height +0.865× sitting height. This further clarified that we could estimate the length of nasogastric tube insertion based on the formula. At the same time, this formula has a certain guiding significance for early calculation of the required length of the catheter for patients, and provides a new reference method for clinical evaluation.

The shortcoming of this study was that the sample size in this study was relatively small, and the formula was not further verified. Therefore, this formula needs to be further corrected. In the future, researchers can conduct multi-center and large sample verification of the correction formula, and achieve individualized nursing operation, thereby improving the therapeutic effect of gastric tube indwelling.

## Conclusion

In summary, height, sitting height, gender, BMI and the length from earlobe to xiphoid process are the factors affecting NCL. There is a significant positive correlation between height and sitting height and NCL. On this basis, the length of nasogastric tube insertion can be estimated.

## Data Availability

The original contributions presented in the study are included in the article/Suplementary Material, further inquiries can be directed to the corresponding authors.
